# The transcriptional signature of human ovarian carcinoma macrophages is associated with extracellular matrix reorganization

**DOI:** 10.18632/oncotarget.12180

**Published:** 2016-09-21

**Authors:** Florian Finkernagel, Silke Reinartz, Sonja Lieber, Till Adhikary, Annika Wortmann, Nathalie Hoffmann, Tim Bieringer, Andrea Nist, Thorsten Stiewe, Julia M. Jansen, Uwe Wagner, Sabine Müller-Brüsselbach, Rolf Müller

**Affiliations:** ^1^ Institute of Molecular Biology and Tumor Research (IMT), Center for Tumor Biology and Immunology (ZTI), Philipps University, Marburg, Germany; ^2^ Clinic for Gynecology, Gynecological Oncology and Gynecological Endocrinology, Center for Tumor Biology and Immunology (ZTI), Philipps University, Marburg, Germany; ^3^ Genomics Core Facility, Center for Tumor Biology and Immunology (ZTI), Philipps University, Marburg, Germany

**Keywords:** tumor-associated macrophages, resident peritoneal macrophages, macrophage polarization, transcriptional signature, extracellular matrix

## Abstract

Macrophages occur as resident cells of fetal origin or as infiltrating blood monocyte-derived cells. Despite the critical role of tumor-associated macrophages (TAMs) in tumor progression, the contribution of these developmentally and functionally distinct macrophage subsets and their alteration by the tumor microenvironment are poorly understood. We have addressed this question by comparing TAMs from human ovarian carcinoma ascites, resident peritoneal macrophages (pMPHs) and monocyte-derived macrophages (MDMs). Our study revealed striking a similarity between TAMs and pMPHs, which was considerably greater that the resemblance of TAMs and MDMs, including their transcriptomes, their inflammation-related activation state, the presence of receptors mediating immune functions and the expression of tumor-promoting mediators. Consistent with these results, TAMs phagocytized bacteria, presented peptide antigens and activated cytotoxic T cells within their pathophysiological environment. These observations support the notion that tumor-promoting properties of TAMs may reflect, at least to some extent, normal features of resident macrophages rather than functions induced by the tumor microenvironment. In spite of these surprising similarities between TAMs and pMPHs, bioinformatic analyses identified a TAM-selective signature of 30 genes that are upregulated relative to both pMPHs and MDMs. The majority of these genes is linked to extracellular matrix (ECM) remodeling, supporting a role for TAMs in cancer cell invasion and ovarian cancer progression.

## INTRODUCTION

High-grade serous ovarian carcinoma (HGSC) is the most common ovarian malignancy with a dire prognosis with an overall 5-year survival rate of <40% [1]. The features that contribute to the fatal nature of ovarian HGSC and distinguish this cancer from other human malignancies include the peritoneal environment, which is frequently formed by the effusion building up in the peritoneal cavity. This malignancy associated ascites is rich in tumor-promoting soluble factors [2] and immune cells, in particular tumor-associated T cells (TATs) [3] and tumor-associated macrophages [4, 5] (TAMs).

TAMs play a crucial role in promoting tumor cell proliferation, dissemination, chemoresistance and immune evasion, as suggested by the correlation of disease progression with macrophage density in different types of human cancer and mouse models, including ovarian HGSC [6–8]. Although TAMs can be derived from recruited blood monocytes [9–11], more recent evidence clearly points to a substantial contribution by tissue-resident macrophages [12–18].

A hallmark of macrophages is their plasticity in response to their microenvironment [19], with “M1” and “M2” macrophages as operationally defined extremes [20]. Classical M1 activation confers immune stimulatory, pro-inflammatory properties, while alternatively activated M2 macrophages comprise a wide spectrum of subtypes with functions in tissue repair, angiogenesis and immune regulation. TAMs have been proposed to resemble “M2” macrophages, in agreement with their role in tumor promotion and immune suppression. Consistent with this conclusion, expression of the classical M2 marker CD163 on TAMs showed a strong correlation with early relapse of serous ovarian carcinoma after first-line therapy [4]. Furthermore, data derived from mouse models showed that pro-inflammatory signaling pathways are defective in TAMs [7, 20–23]. However, macrophages can also adopt properties of both M1 and M2 cells [19], and several studies suggest that TAMs represent such a mixed-polarization phenotype [4, 11, 20, 24].

Macrophages in the adult mouse can have two developmentally different origins. While infiltrating macrophages are derived from blood monocytes produced by the bone marrow, tissue macrophages, including alveolar, peritoneal, splenic, hepatic (Kupfer cells) and dermal (Langerhans cells) macrophages, are of fetal (yolk sac) origin [17, 25–30]. The transcription factor MYB is essential for the development of murine bone-marrow macrophages [25], whereas GATA6 is indispensable for the fetal lineage and distinguishes resident from infiltrating macrophage [26, 31]. Whether ovarian cancer ascites-associated macrophages are derived from infiltrating monocytes, resident peritoneal macrophages or both is unclear.

Our current view of the tumor-mediated activation state of macrophages is largely based on studies comparing TAMs to monocyte-derived macrophages (MDMs) [9, 32]. Systematic analyses comparing TAMs to normal, uncultured macrophages are currently not available. The present study reveals for the first time a surprising similarity between TAMs and resident peritoneal macrophages (pMPHs) with respect to both their differentiation and polarization state, but also delineates a TAM-selective signature associated that is associated with extracellular matrix remodeling.

## RESULTS

### Similar expression of differentiation and activation markers by TAMs and pMPHs

**Figure 1:**
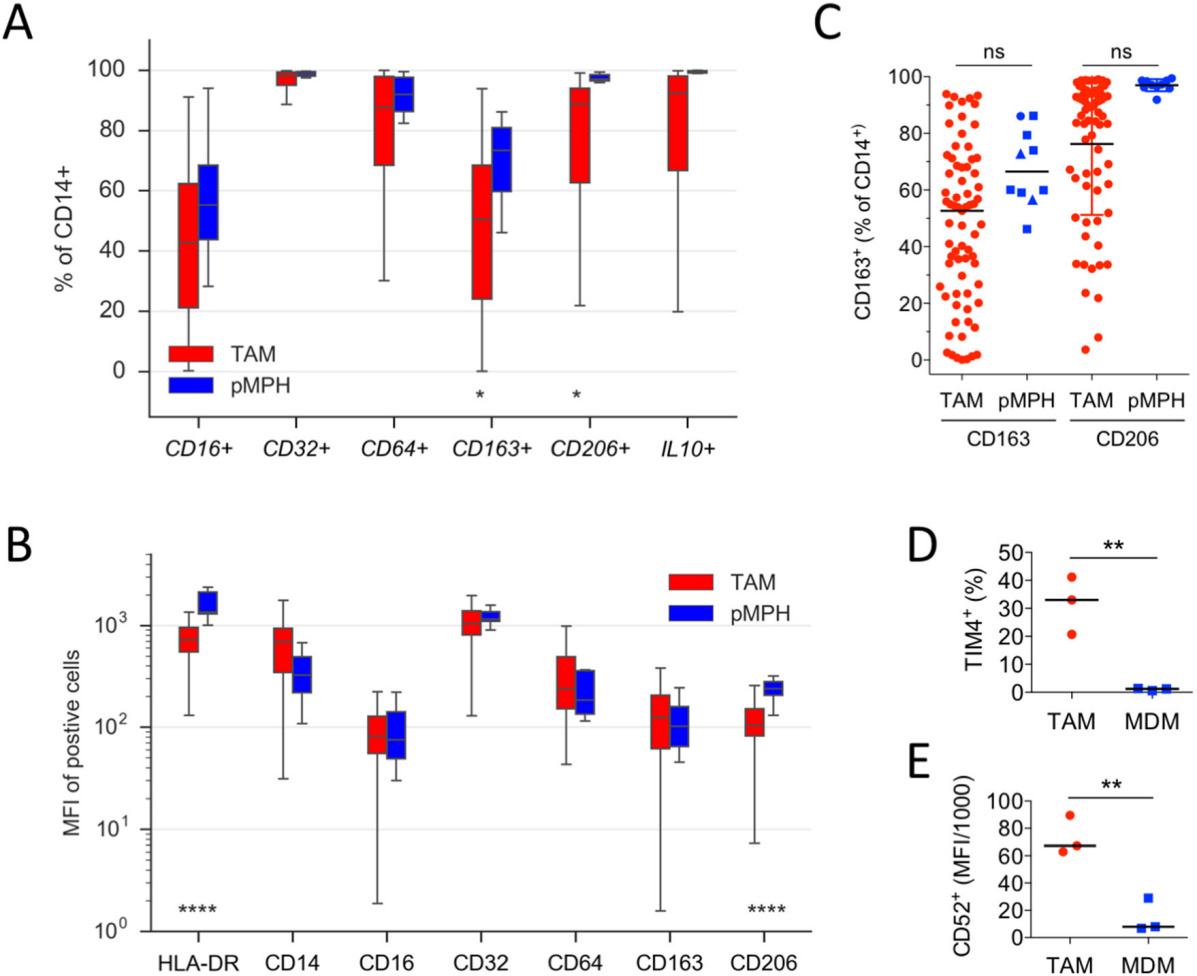
Similarities of TAMs and pMPHs **A, B.** Flow cytometry analysis of freshly isolated TAMs and MPHs for cell surface receptor and intracellular IL-10 expression. The data show the fraction of CD14^+^ cells (A) or the median fluorescence intensity (MFI) of positive cells (B). Sample sizes were n=71 (TAM) and n=10 (pMPH), respectively. **C.** Quantification of CD163^+^ and CD206^+^ cells in TAM (n=71) and pMPH (n=10) samples isolated from patients undergoing surgery for myomatosis (squares), ovarian cysts (triangles) or endometriosis (circle). **D, E.** Flow cytometry analysis of TIMD4 (% positive) and CD52 (MFI) on TAMs (n=3) and MDMs (n=3). *p<0.05; **p<0.01; ****p<0.0001 by *t*-test; ns: not significant; horizontal lines: median.

We first compared pMPHs from patients undergoing hysterectomy for non-malignant diseases and TAMs from ovarian cancer ascites (Supplementary Table S1) for expression of inflammation and activation markers by flow cytometry. The data in Figure 1A and 1B show that surface expression of the Fcγ receptors CD16 (FCGR3), CD32 (FCGR2) and CD64 (FCGR1) was similar for both cell types, with respect to both the fraction of positive cells (Figure 1A) and the mean fluorescence intensity (MFI; Figure 1B). HLA-DR was expressed on >95% of all cells analyzed, but the measured MFI was clearly higher on MPHs (Figure 1B). The “M2” markers CD163, CD206 and intracellular IL-10 were similarly expressed by both TAMs and pMPHs, except for a tendency towards a higher fraction of CD163^+^ and CD206^+^ in MPH samples (Figure 1B). Our data also indicate that neither CD163 nor CD206 distinguishes TAMs from pMPHs, regardless of the underlying non-malignant condition of the patients (Figure 1C). Consistent with our observation, human pMPHs have previously been shown to express high levels of CD163 and display characteristics of anti-inflammatory macrophages [33]. Thus, while there are detectable differences between TAMs and pMPHs, both cell types do not differ in terms of a directional inflammation-related polarization switch.

**Figure 2:**
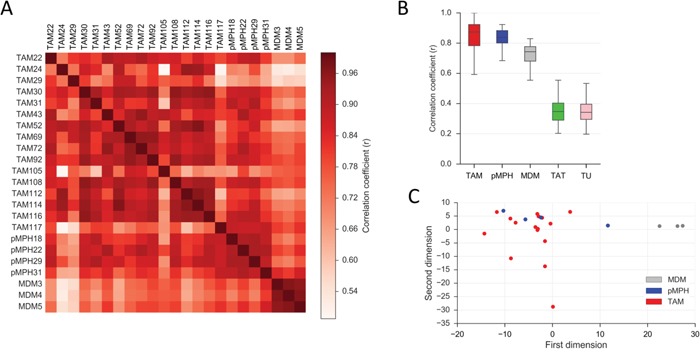
Similarity of TAMs and pMPH transcriptomes **A.** Correlation heatmap (Pearson r) of the transcriptomes of TAM, pMPH and MDM samples. **B.** Pearson correlation (r) of the TAM transcriptome to that of pMPHs, MDMs, TATs and tumor cells (TU) for all individual samples. Bars: 95% CI; horizontal lines: median. **C.** Principle component analysis (PCA) of TAM, pMPH and MDM samples. Sample sizes were n=16 (TAM), n=4 (pMPH), n=3 (MDM), n=5 (TAT) and n=19 (TU), respectively, in all panels.

To identify differences between TAMs and pMPHs by a systematic approach we compared the transcriptome of 17 TAM, 4 pMPH and 3 of non-polarized (M0) MDM samples by RNA sequencing (all RNA-Seq data in Supplementary Dataset S1; TAM and pMPH samples were uncultured primary cells). Pearson correlation of median gene expression values showed a high similarity of all TAM and pMPH transcriptomes (r = 0.93), while MDM were considerably more divergent (r = 0.79; Supplementary Figure S1). Pearson correlation analysis for individual samples yielded a similar result (median r = 0.84 for TAMs versus pMPHs; r = 0.74 for TAMs versus MDMs; Figure 2A, 2B). These results were confirmed by PCA which split our samples in two groups: TAM/pMPH and MDM (Figure 2C). As expected the correlation between TAMs and TATs or tumor cells was very low (r = 0.34; Figure 2B).

**Figure 3:**
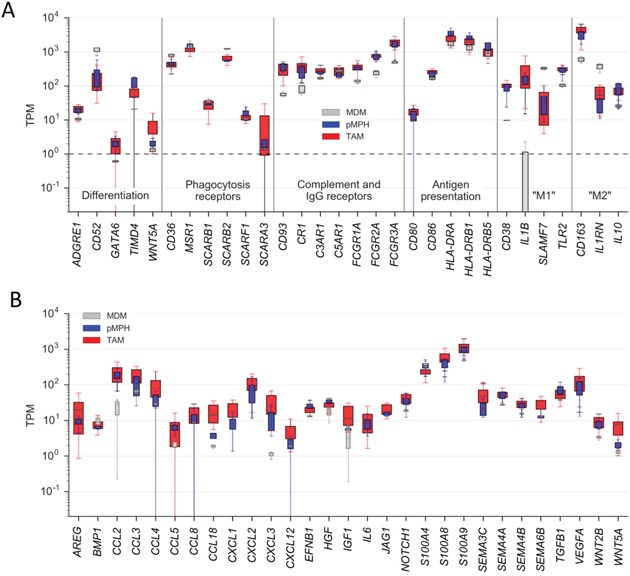
Expression of genes coding for proteins with immune or pro-tumorigenic functions by TAMs and normal macrophages **A.** Expression of genes coding for differentiation markers (resident/infiltrating macrophages), immune functions or “M1/M2” polarization markers (RNA-Seq data). **B.** Expression of genes associated with pro-tumorigenic functions. Boxes show the upper and lower quartiles, whiskers the 95% confidence intervals amd horizontal lines the median. Sample sizes were n=16 (TAM), n=4 (pMPH), n=4 (MDM), respectively. *p<0.05; **p<0.01; ****p<0.0001 by *t*-test; ns: not significant.

Consistent with the global resemblance of TAMs and pMPHs, at least 3 markers selectively expressed in resident macrophages in the mouse [26–29, 31, 34–36], i.e., *ADGRE1* (F4/80), *GATA6* and *TIMD4*, were expressed at similar levels in both TAMs and pMPHs, but much lower, if at all, in MDMs (Figure 3A). The opposite scenario was observed for *CD52*, reported to be preferentially expressed in monocyte-derived cells [37]. In agreement with these data, TIMD4 surface expression was stronger on TAMs compared to MDMs (Figure 1D), whereas CD52 was higher on MDMs (Figure 1E).

The RNA-Seq data also revealed similar expression levels in TAMs and MPHs for all markers of macrophage functions tested, including phagocytosis-associated receptor genes *(CD36, MSR1, SCAR* family genes, *TIMD4, CD163), FCGR* genes, complement receptor genes (*CD93/C1Q-R1, C3AR, CR1, C5AR1*) and all polarization marker genes tested, including *CD163* and *IL10* (Figure 3A). These observations are in perfect agreement with the flow cytometry analysis described above (Figure 1A–1C). Similar observations were made for genes encoding pro-tumorigenic cytokines or growth factor (Figure 3B), previously found to be mainly expressed by TAMs within the ovarian cancer microenvironment [38]. These results indicate that ovarian carcinoma ascites-associated TAMs closely resemble pMPHs not only with respect to their activation state but also with regard to some of their pro-tumorigenic functions.

### Immune functions of TAMs

The similarity with pMPHs described above suggested that macrophage-mediated immune functions might be preserved in ovarian carcinoma TAMs, at least to some extent. While TAMs can be maintained ex vivo for functional assays under conditions resembling their pathophysiological microenvironment (ascites), it is not possible to culture pMPHs under physiological conditions (e.g, peritoneal fluid). We therefore focused our analyses on short-term cultures of TAMs in ascites, and used MDMs as positive controls.

**Figure 4:**
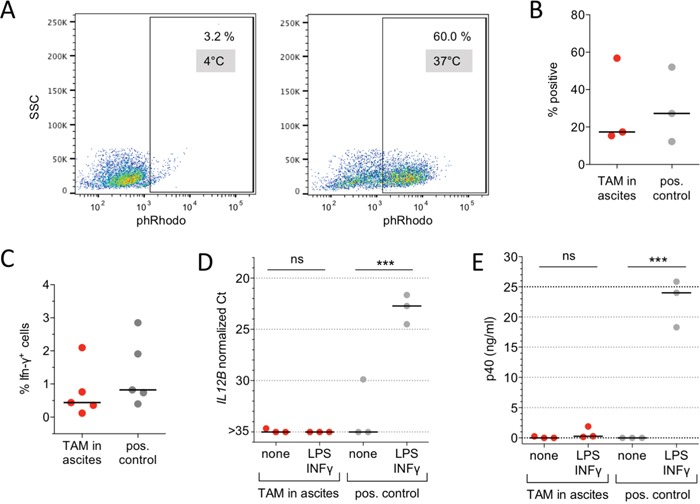
Immune functions of ovarian carcinoma TAMs **A.** Phagocytosis of E. coli particles conjugated to a pH-sensitive fluorochrome (pHrodo) by ovarian cancer TAMs in ascites. The plots show flow cytometry analysis of cells incubated at 37°C (active phagocytosis) and 4°C (background control). **B.** Quantification of 3 independent experiments as in panel A with TAMs in ascites. MDMs in RPMI medium were included as positive control. **C.** Antigen-specific CD8^+^ T cell stimulation. TAMs from the ascites of 5 ovarian cancer patients cultured in ascites were loaded with the recall antigen peptide mix CEFT and analyzed for their ability to stimulate INFγ production by co-cultured T cells. The fraction of CD8^+^IFNγ^+^ cells was determined by flow cytometry. MDMs established from 5 different donors were used as positive control. **D.** *IL12B* expression in TAMs (n=3) cultured in autologous ascites for 2 d. Cultures were stimulated with LPS (100 ng/ml) and INFγ (20 ng/ml) or solvent only (none) for 24 h and RNA was analyzed by RT-qPCR. MDMs (n=3) in RPMI were included as positive control. **E.** p40 (IL-12B/IL-23) protein concentrations in the culture medium of the experiments in panel D. Each dot represents an independent sample in B-E. Horizontal lines: median; vertical bars: range. ***p<0.001 by *t*-test between unstimulated and INFγ/LPS-stimulated cells in panels D and E; ns: not significant.

An essential function of tissue resident macrophages is the phagocytosis of pathogens and apoptotic cells [27, 33, 39–41]. Consistent with the expression pattern of phagocytosis-associated receptors (Figure 3A) the TAMs were able to efficiently phagocytize labelled *E. coli* particles (Figure 4A, 4B).

Another function reported for human pMPHs is the presentation of peptide antigens [42–46]. In view of the high expression of *HLA* genes in TAMs (Figure 1) we therefore investigated the capacity of TAMs to trigger antigen-specific cytotoxic T cell activation. For this purpose, we exposed TAMs to a mixture of antigens (CEFT) derived from pathogens most individuals have previously been sensitized to and have developed antigen-specific memory T cells. Restimulation with these recall antigens results in the activation of this subset of antigen-specific T cells (1% of all T cells). Using intracellular IFNγ production as an activation marker, we found a clear stimulation of CD8^+^ T cells by TAMs within a range similar to the positive control (Figure 4C; non-stimulated cells served as negative controls for background substraction). Collectively, these data show that known immune functions of pMPHs are retained by TAMs in their pathophysiological environment.

Previous studies in mouse models have shown that pro-inflammatory signaling pathways are non-functional in TAMs in different tumor types [7, 20–23], and that ovarian cancer TAMs are refractory to pro-inflammatory stimuli [47]. In agreement with these observations we found that both expression of the pro-inflammatory mediator gene *IL12B* and secretion of its product p40 are not inducible by lipopolysaccharide (LPS) and interferon-γ (INFγ) in TAMs, whereas a strong induction was observed with the positive control (Figure 4D, 4E).

### Genome-wide expression profiles of TAMs and pMPHs and delineation of a TAM-specific signature

**Figure 5:**
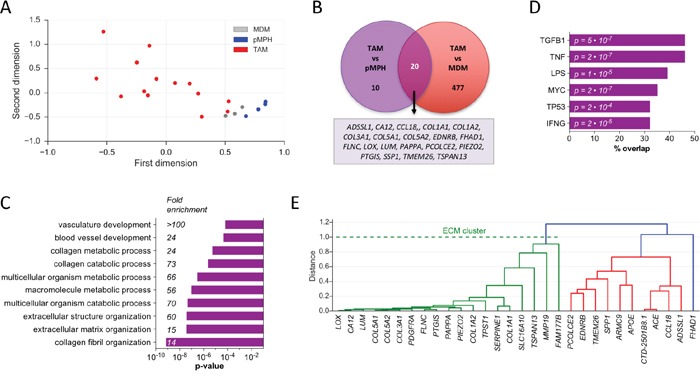
Identification of a transcriptional ECM signature of genes upregulated in TAMs versus normal macrophages **A.** PCA of TAM, pMPH and MDM samples for the upregulated gene set. TAM52 (x=3.4) is outside the range displayed. **B.** Venn diagram of genes upregulated in TAMs versus pMPHs or MDMs (FC >3; TPM>1.5). **C.** Functional annotation of upregulated genes by GO term analysis (Supplementary Table S3). The bar plot shows the top 5 terms (p< 0.001). **D.** Upstream regulator analysis (Ingenuity pathway database) of upregulated genes with a minimum overlap of gene sets of 30% (query gene set and genes targeted by indicated pathways). **E.** Correlation-based hierarchial clustering of upregulated genes. See Materials and Methods for details.

We next sought to gain further insight into the specific phenotype of ovarian cancer TAMs by in-depth analysis of the transcriptomic data. Toward this end, we started out by analyzing the RNA-Seq data sets with edgeR, a Bioconductor package specifically developed for reliable gene-specific dispersion estimation in small samples by ranking genes that behave consistently across replicates more highly than genes that do not [48, 49]. The edgeR tool identified a group of 21 genes expressed at significantly different levels in TAMs versus pMPHs (Supplementary Table S1). We then searched for genes showing highly correlated expression pattern across all TAM samples (r >0.9) and a higher median expression in TAMs versus pMPH or vice versa (FC >3-fold). This resulted in the definition of an extended datasets of 30 genes upregulated in TAMs (Supplementary Dataset S2; Supplementary Figure S2; Figure 5A). PCA of TAM, pMPH and MDM samples for the upregulated gene set yielded clearly separable clusters for TAMs versus pMPHs or MDMs (Figure 5A), showing that the chosen strategy was successful.

We performed similar analyses with TAMs and MDMs (Supplementary Table S2) leading to an extended datasets of 497 upregulated genes (Figure 5B). The majority of genes upregulated in TAMs versus pMPHs (20/30) were also upregulated relative to MDMs (Figure 5B), thus providing a further validation of the upregulated gene set. Since only few genes were downregulated in TAMs versus pMPHs (n = 4; Supplementary Dataset S3; Supplementary Figure S3 and Supplementary Figure S4), we focused all further analyses on the upregulated gene set.

### A TAM-specific ECM gene cluster

Gene Ontology (GO) term analysis showed a very strong association of the upregulated genes with extracellular matrix (ECM) and collagen fibril organization (Figure 5C; Supplementary Table S3). IPA Upstream Regulator Analysis identified these genes as targets mainly of TGFB and pro-inflammatory (LPS, TNF, INFG) signaling pathways (Figure 5D). This is intriguing in light of previous studies reporting the presence of TNFα and TGFβ1 in the ascites of the vast majority of ovarian cancer patients and their association with disease progression [2, 4, 50–53]. Hierarchial clustering using correlation as distance metric identified a group of 19 co-regulated genes, which make up 63% of all upregulated genes identified (Figures 5E and Supplementary Figure S2). Intriguingly, this cluster harbors virtually all regulated genes associated with ECM remodeling. We subsequently refer to these genes as the “ECM cluster”.

**Figure 6:**
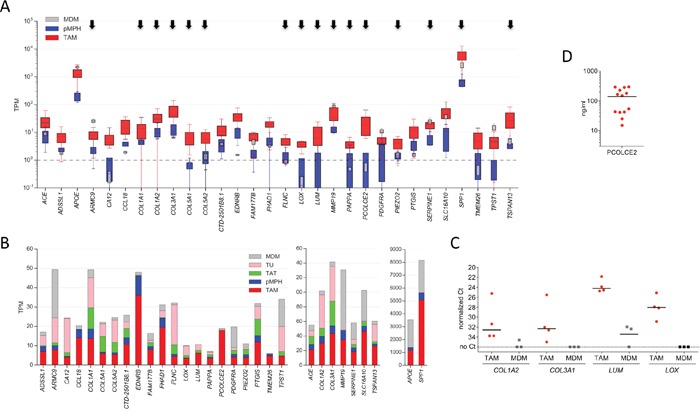
Expression of ECM signature genes **A.** Expression of upregulated genes across TAM, pMPH and MDM samples. Data are represented as in Figure 2. Arrows point to genes with functions in ECM remodeling. **B.** Expression of upregulated genes (panel B) in different ovarian carcinoma-associated cell types, pMPHs and MDMs. The stacked boxes show the respective median expression values (TPM). **C.** Validation of RNA-Seq data by RT-qPCR. Each symbol represents a biological replicate (TAM: n=4; MDM: n=3). Horizontal lines: median. **D.** Concentrations of PCOLCE 2 in ascites from ovarian HGSC patients determined by ELISA (n=10).

The expression patterns of the ECM signature genes in TAMs and pMPHs shown in Figure 6A (red versus blue bars) clearly document their selective upregulation in TAMs. Similar results were obtained when expression in TAMs was compared to MDMs (red versus grey bars in Figure 6A) with only few exceptions, providing further evidence tor the robustness of the ECM signature and its association with tumor-triggered events. Comparison with tumor cells and TATs showed that most of these genes are mainly expressed by TAMs and tumor cells (Figure 6B). We also analyzed several genes of the ECM signature by RT-qPCR and could fully verify the RNA-Seq data in all for cases (Figure 6C). Finally, we also found readily detectable levels of PCOLCE2 protein by ELSIA in ovarian cancer ascites (Figure 6D), supporting a potential functional relevance of the upregulated ECM signature genes.

Contamination of TAM samples with tumor cells was very low in most samples, in several cases even undetectable (Supplementary Table S4). Furthermore, none of the ECM cluster genes were expressed at substantially higher levels by tumor cells relative to TAMs (Figure 6B), thus ruling out the possibility that the expression observed in TAM samples results from residual tumor cells. Another cell type present in ascites, albeit at low numbers, are carcinoma-associated fibroblasts (CAFs) [54]. Importantly, all CAF marker genes analyzed were either expressed at similar levels in both TAMs and pMPHs (Supplementary Figure S5) and/or did not show any appreciable correlation with expression of ECM cluster genes, as exemplified for COL3A1 in Supplementary Figure S6. Similar results were obtained for markers of mesenchymal stem cells (MSCs) and mesothelial cells (Supplementary Figure S5 and Supplementary Figure S7), known to be present ovarian cancer ascites in ascites [54]. We therefore conclude that potential contaminations of TAM samples do are unlikely to make a significant contribution to the observed TAM-specific signature.

Importantly, proteins encoded by upregulated genes are also found in the ascites fluid from ovarian cancer patients, supporting potentially relevant functions. This is exemplified in Figure 5C for PCOLCE 2 (upregulated in TAMs ~20-fold). Furthermore, previous proteomic profiling of ovarian cancer ascites identified several proteins relevant in this context, including multiple collagens and lumican [55]. In addition, collagen type I, III and IV fragments have been found at elevated levels in serum samples from ovarian cancer patients [56].

Taken together, these observations suggest that the upregulation of ECM remodeling genes is a hallmark of ovarian cancer TAMs. The coordinate regulation of the genes within this cluster is presumably caused by a tumor-triggered signaling pathway rather than merely a consequence of a genomic co-localization. The 5 *COL* genes of the ECM cluster, for instance, are localized on 4 different human chromosomes (2, 7, 9 and 17), *LUM* on chromosome 12 and *PCOLCE2* on chromosome 3 (Ensembl).

## DISCUSSION

### Activation state and immune functions of TAMs

Our flow cytometry and transcriptome data clearly show that markers are expressed in ovarian cancer TAMs in a way inconsistent with a directional inflammation-related polarization. On the other hand, these analyses revealed a surprisingly high similarity of TAMs and pMPHs, including their activation state and the expression of molecules with essential roles in immune functions. Thus, tissue resident macrophages, like TAMs, are characterized by a high expression of the alternative activation markers CD163 and CD206 [33]. and both TAMs and pMPHs express genes with essential functions in phagocytosis or antigen presentation at similarly high levels (Figures 1A–1C, Figure 3A).

Consistent with the high expression of scavenger receptors and other molecules involved in phagocytosis (Figure 3A), TAMs efficiently phagocytosed bacteria within their pathophysiological environment, i.e., ovarian cancer ascites (Figure 4A, 4B). TAMs share this function with pMPHs, known as major players in the clearance of pathogens and damaged cells [33]. Furthermore, in agreement with the strong expression of multiple HLA genes (Figure 3A), TAMs were able to trigger an antigen-specific cytotoxic T cell response (Figure 4C), which is also known as a function of pMPHs [42–45].

Previous work has shown that peritoneal macrophages in the mouse consist of two functionally and developmentally distinct subsets, with cells of fetal origin representing the vast majority [30]. In the mouse, these resident cells differ from infiltrating monocyte-derived macrophages by the specific expression of several markers, including *ADGRE1* (F4/80), *GATA6* and *TIMD4* [26–29, 31, 34–36]. Our data show that human pMPHs also express much higher levels of these marker genes than MDMs (Figure 1D, 1E), suggesting that these markers may also be applicable to human cells. TAMs and pMPHs showed very similar expression patterns of these markers, consistent with the hypothesis that pMPHs are a major origin of TAMs. However, it cannot be ruled out that infiltrating monocytes are converted to TAMs resembling pMPHs by tumor-borne mediators.

Previous work has identified TAMs as the major source of a number of pro-tumorigenic or immune suppressive protein mediators within the ovarian cancer microenvironment [38], The data presented here show that the corresponding genes are expressed at similar levels in TAMs and pMPHs, while their expression is lower in MDMs in most cases (Figure 2D). It is therefore likely that some pro-tumorigenic effects mediated by TAMs reflect functions of pMPHs rather than tumor-induced alterations.

Our data also confirm the previously described refractoriness of TAMs to inflammatory stimuli [7, 20–23, 47], exemplified by the unresponsiveness of the *IL12B* gene to LPS and INFγ in ovarian cancer TAMs (Figure 4D, 4E). Since pMPHs are principally inducible by pro-inflammatory stimuli (Figure 4D, E) and the induction of proinflammatory genes in MDMs is repressed by ascites (as shown for *IL12B* in Supplementary Figure S8), it is likely that the observed lack of TAMs to LPS and INFγ is caused by the tumor microenvironment. This suggests that ovarian cancer ascites affects macrophage functions to varying degrees, with phagocytosis and antigen presentation remaining intact and inflammatory responses being suppressed. The molecular mechanisms underlying this repression remain obscure, as the comparative RNA-Seq data did not provide insights into the transcriptional signaling pathways affected.

### Upregulation of ECM remodeling genes in TAMs

Our study identified an ECM gene cluster as a specific feature of ovarian cancer TAMs (Figures 5 and Figure 6), suggesting that TAMs figure in collagen deposition, fibrillogenesis and ECM remodeling. In this context it is noteworthy that fibrillar collagen has been shown to enhance the invasive properties of tumor cells by accelerating their movement along these fibers and macrophages clearly enhance cancer cell invasion [6, 24]. Macrophages are also indispensable for mouse mammary gland development owing to their critical function in promoting collagen fibrillogenesis [57].

A number of published observations have linked the products of several of the ECM cluster genes to macrophage-mediated matrix remodeling and cancer cell invasion [24]. Apart from the collagens, other proteins encoded by the ECM cluster with instrumental functions in matrix deposition and remodeling include (i) lumican (LUM), which regulates collagen fibril organization and growth [58, 59], (ii) lysyl oxidase (LOX) with crucial functions in the cross-linking of ECM proteins [60] and (iii) procollagen C-endopeptidase enhancer 2 (PCOLCE2), which promotes the enzymatic cleavage of type I procollagen to yield mature structured fibrils [61, 62]. Importantly, PCOLCE2 protein was detectable at appreciable levels in the ascites of ovarian cancer patients (Figure 6D).

### Clinical relevance of ECM remodelling

On the basis of our observations it is tempting to speculate that TAMs support tumor cell adherence and invasion by secreting ECM remodeling proteins. Such a scenario is indeed strongly supported by a mouse model of transcoelomic ovarian cancer dissemination, which showed a clear dependence of peritoneal colonization on macrophage-mediated effects on the ECM through metalloproteinase 9 [63]. Furthermore, other researchers showed that macrophage depletion in mice resulted in decreased ascites formation and peritoneal colonization [64–66].

Tumor cell spheroids from ovarian cancer ascites can adhere to, disintegrate and spread on ECM components [67, 68], suggesting that the macrophage-triggered reorganization of collagen deposition may promote ovarian cancer cell invasion. This result is consistent with previous observations associating ECM remodeling genes with a poor clinical course of ovarian cancer [51, 69–73]. For example, Cheon et al [69]. described a relapse-associated signature that consists of genes coding for collagen/ECM remodeling proteins. Intriguingly, this signature is regulated by TGFβ1 signaling as predicted for the ECM cluster identified in the present work (Figure 4D).

Busuttil and colleagues [71] identified a “stromal-response” signature in ovarian cancer that is associated with poor survival and enriched for genes encoding inflammatory and extracellular matrix proteins expressed by the tumor-associated stroma. Furthermore, the mesenchymal subtype of ovarian HGSC, characterized by the upregulation of ECM remodeling genes, has the worst clinical outcome of all subtypes [72, 73]. Our observations extend these findings by providing compelling evidence that genes associated with ECM restructuring are coordinately upregulated in ovarian cancer TAMs. This may explain, at least in part, the critical role of macrophages in ovarian cancer progression [4, 74].

## MATERIALS AND METHODS

### Patient samples

Clinical samples (Supplementary Table S5) were obtained from untreated patients undergoing surgery for ovarian carcinoma (mostly HGSC) or hysterectomy for non-malignant diseases lacking peritoneal effusions. Informed consent was obtained from all patients according to the protocols approved by the local ethics committee. All experiments were conducted in agreement with the Helsinki declaration.

### Isolation and culture of primary immune cells

Macrophages were isolated from ascites (TAMs) or peritoneal lavage fluids (pMPHs) by density gradient centrifugation (Lymphocyte Separation Medium 1077; PromoCell) and subsequent enrichment on magnetic CD14 microbeads (Miltenyi Biotech). Tumor cells and CD3^+^ T cells were isolated as described [38]. MDMs were generated from monocytes (6-day differentiation for RNA experiments, 10-day cultures for flow cytometry) from healthy donors as described [75] and in RPMI with human AB serum.

### Flow cytometry analysis of macrophages

TAMs from malignant ascites or pMPHs from peritoneal lavage fluid were stained with FITC-labeled anti-CD14 (Miltenyi Biotech), APC-labeled anti-CD206 (BioLegend), APC-labeled anti-HLA-DR or APC-labeled anti-CD206 (Biozol), PE-labeled anti-CD163, PE-labeled anti-CD64, PE-Cy7-labeled anti-CD16 and APC-labeled anti-CD32 (eBioscience) as described previously [4]. Intracellular staining was performed with PE-labeled anti-IL-10 (BD Biosciences) after permeabilization for 20 min at 4 C using BD Cytofix Cytoperm Plus Fixation Permeabilization Kit (BD Biosciences). Additionally, APC-labeled anti-CD52 or APC-labeled anti-TIMD4 (Biolegend) was used for surface staining of TAMs and MDMs from healthy donors. Isotype control antibodies were from BD Biosciences, Miltenyi Biotech and eBioscience. Cells were analyzed by flow cytometry and results were calculated as percentage of positive cells and mean fluorescence intensities (MFI).

### ELISA of ascites

Concentrations of PCOLCE 2 in ascites from ovarian cancer patients were determined using an ELISA Kit from Biozol according to the instructions of the manufacturer.

### T cell activation

Antigen-specific T cell activation by macrophages was determined essentially as described [75]. In brief, MDMs or TAMs were loaded with 1μg/ml CEFT peptide pool (jpt Peptide Technologies, Berlin, Germany) as recall antigens and incubated with a 5-fold excess of lymphocytes for 18 h in the presence of Brefeldin A (Sigma Aldrich, Steinheim, Germany). Lymphocytes were harvested and stained with anti-CD8-APC (Miltenyi Biotec, Bergisch Gladbach, Germany) and after permeabilization with anti-IFNγ-FITC (eBioscience, Frankfurt, Germany). Flow cytometry (FACS Canto, BD Bioscience, Heidelberg, Germany) data were expressed as IFNγ+/CD8+ cells after subtracting background staining of non-stimulated controls.

### Analysis of phagocytosis

Phagocytotic capacity was determined by incubating macrophages with pHrodo® Red E. coli BioParticles conjugate (Thermo Fisher) for 15 min in R10AB medium and subsequent quantification by flow cytometry.

### RT-qPCR

Isolation of total RNA and RT-qPCR were carried out as described [76], using the following primers:

RPL27_fw: 5′ AAAGCTGTCATCGTGAAGAAC

RPL27_rv: 5′ GCTGTCACTTTGCGGGGGTAG

IL12B_fw: 5′ GCGAGGTTCTAAGCCATTCG

IL12B_rev: 5′ ACTCCTTGTTGTCCCCTCTG

COL1A2_fw: 5′ AGCTCCAAGGACAAGAAAC ACGTCTGG

COL1A2_rev: 5′ AGGCGCATGAAGGCAAG TTGGGTAG

COL3A1_fw: 5′ CTGGACCCCAGGGTCTTC

COL3A1_rev: 5′ CATCTGATCCAGGGTTTCCA

LOX_fw: 5′ TGGCACAGTTGTCATCAACA

LOX_rev: 5′ TCTTCAAGACAGAAACTT GCTTT

LUM_fw: 5′ TGGAGGTCAATCAACTTGAGAA

LUM_rev: 5′ CCAAACGCAAATGCTTGAT.

### RNA Sequencing (RNA-Seq)

RNA-Seq was performed as described [75]. Sequencing data were deposited at EBI ArrayExpress (E-MTAB-3167, E-MTAB-3398, E-MTAB-4162, E-MTAB-4764). Genome assembly and gene model data were retrieved from Ensembl release 81, hg38. RNA-Seq data were aligned using STAR (version STAR_2.3.1z13_r470) [77]. Gene read counts were established and TPM (transcripts per million) values were calculated as published [75]. Adjustment of RNA-Seq data for contaminating tumor and T cells was performed as describe [38]. Batch effects were removed using Bioconductor tool ComBat [78, 79] after filtering all genes with a variance <1.

### Identification of regulated genes

RNA-Seq data (Supplementary Dataset S1) were filtered for genes with minimum TPM values of 3 and median TPM ratios TAM/TAT >0.1 and TAM/tumor cells >0.1. For the delineation of genes selectively up- or down-regulated in TAMs we applied the Bioconductor package edgeR [48, 49] and identified a group of 21 genes expressed at significantly different levels in TAMs versus pMPHs (FDR = 0.2; Supplementary Table S1). We then used this gene set to identify additional genes showing highly correlated expression patterns across all TAM samples (r >0.9) and no overlaps of TAM and pMPH samples using the upper and lower quartiles, respectively, as thresholds. Upregulated genes were defined by 2-fold higher median TPM values in TAMs versus pMPH, or *vice versa* for down-regulated genes. This resulted in the definition of extended datasets of 30 genes upregulated and 4 genes downregulated in TAMs (Supplementary Dataset S2 and Supplementary Dataset S3). Similar analyses performed for with TAMs and MDMs (Supplementary Table S2) lead to extended datasets of 497 upregulated genes.

### Statistical and bioinformatic analyses

Flow cytometry, ELISA and RT-qPCR data were statistically analyzed by Student's *t*-test (two-sided, equal variance). Results are shown as follows: *p<0.05; **p<0.01; ***p<0.001; ****p<0.0001. Quantiles, confidence intervals and correlation coefficients were calculated using the Python functions *pandas.DataFrame.boxplot ()* and *scipy.stats.pearsonr ()*, respectively. Hierarchical cluster analysis was performed using the scipy.cluster.hierarchy functions *linkage (method=“weighted”, metric=“correlation”)* and *dendrogram ()*. Gene sets were analyzed for *Upstream Regulators* using the Ingenuity Pathway Analysis (IPA) database (Qiagen Redwood City, CA, USA) as described [75]. Functional annotations were performed by gene ontology (GO) enrichment analysis (http://geneontology.org).

## SUPPLEMENTARY FIGURES AND TABLES







## Abbreviations

CI: confidence interval
ELISA: enzyme-linked immunosorbent assay
HGSC: high-grade serous ovarian carcinoma
RNA-Seq: RNA sequencing
TAM: tumor-associated macrophage
TAT: tumor-associated lymphocyte
TPM: transcripts per million.
