# Supplementary material for: The transcriptional signature of human ovarian carcinoma macrophages is associated with extracellular matrix reorganization
Source: Oncotarget. 2016 Sep 21;7(46):75339–52. doi: 10.18632/oncotarget.12180 (PMC5342745; doi:10.18632/oncotarget.12180)
Supplement: Supplementary file 1 [file oncotarget-07-75339-s001.pdf]

The transcriptional signature of human ovarian carcinoma macrophages is associated with extracellular matrix reorganization

SUPPLEMENTARY FIGURES AND TABLES

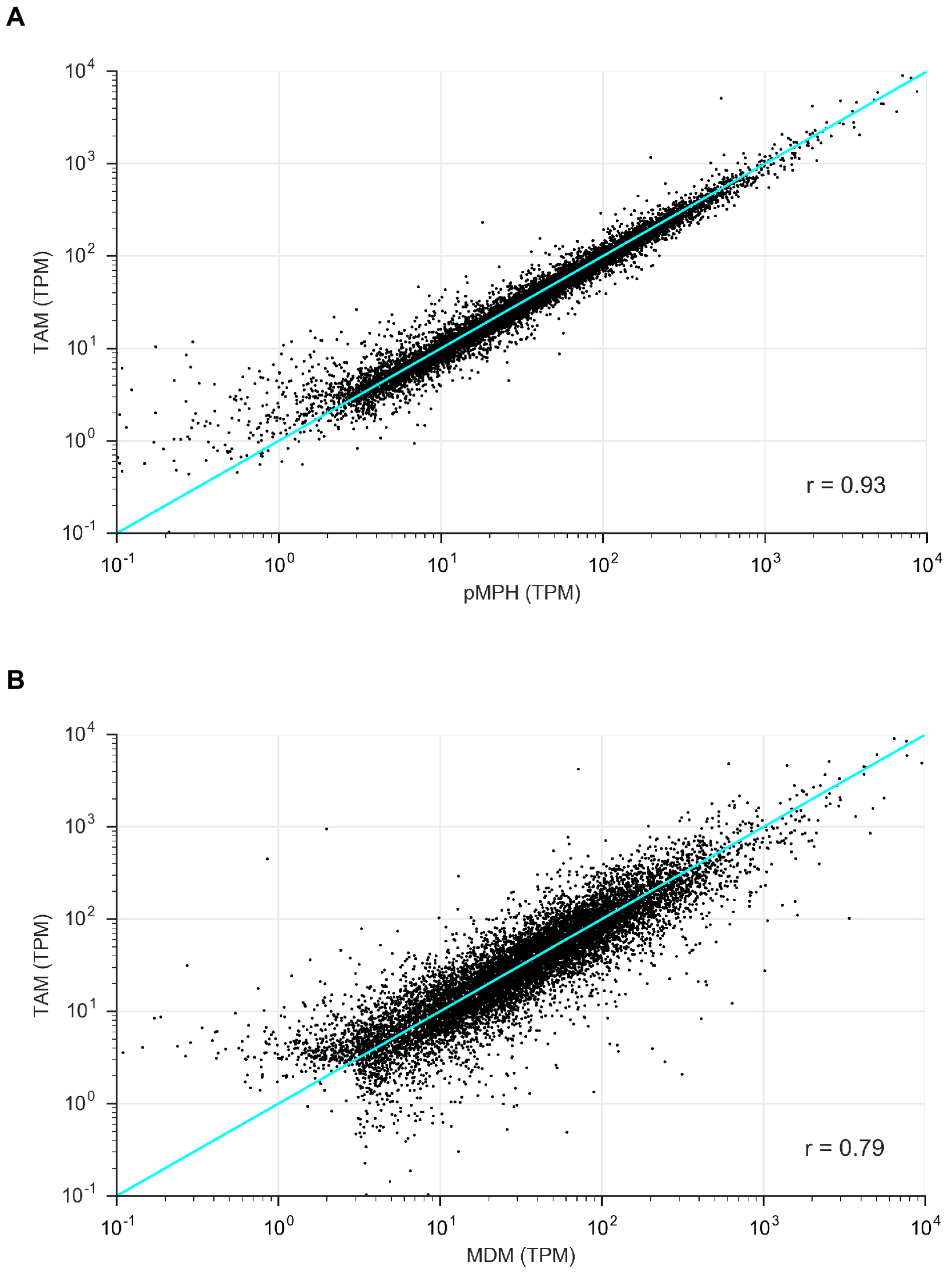

Supplementary Figure S1: Correlation of transcriptomes. A. TAM versus pMPH (median TPM values). **B.** TAM versus MDM. RNA-Seq data were filtered as follows: minimum median TPM = 3.0 in TAM, pMPH or MDM and TPM ratios TAM/TAT >0.10 and TAM/tumor cells >0.10. r: Pearson correlation coefficient.

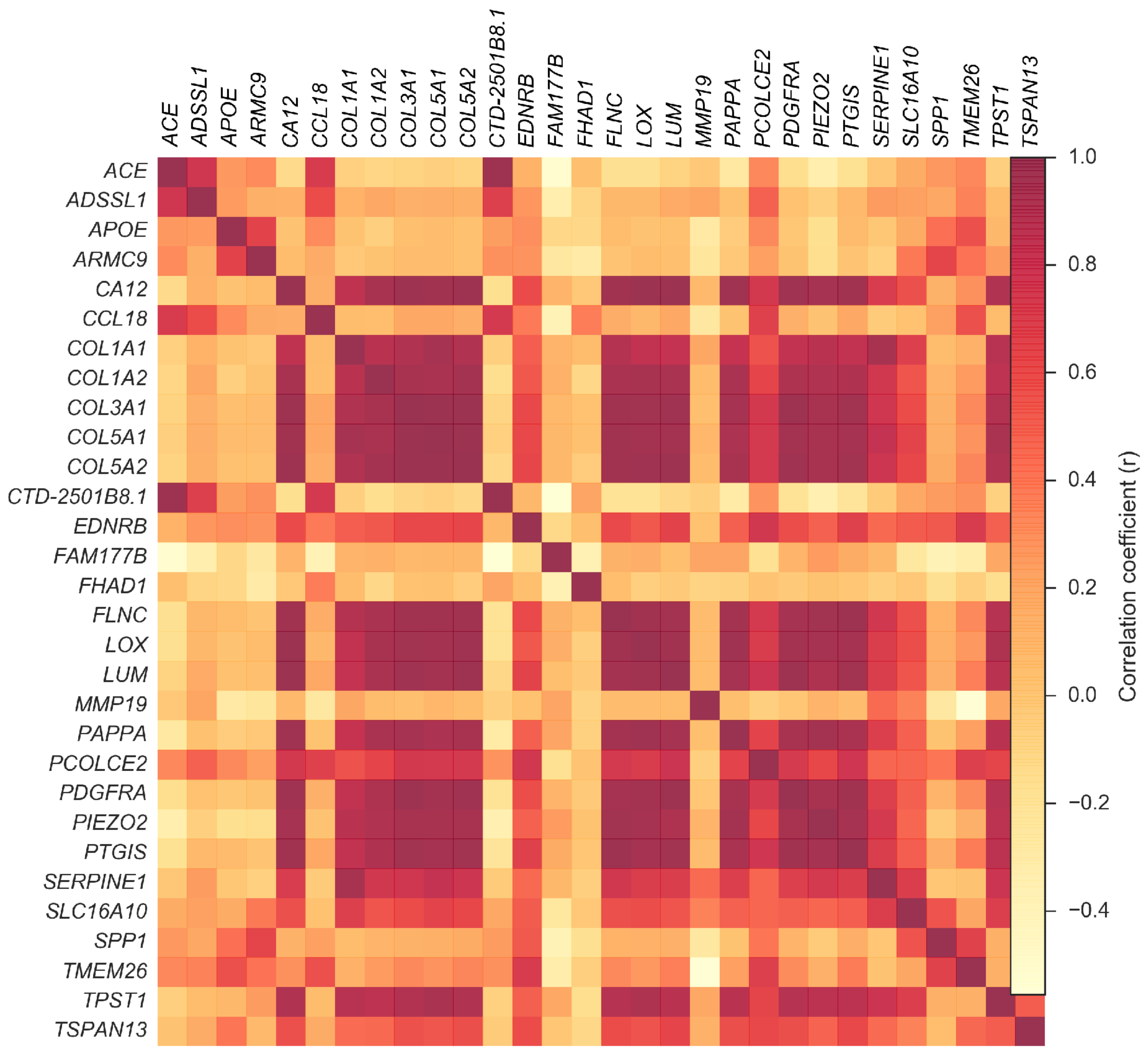

Supplementary Figure S2: Correlation of median TPM values for genes upregulated in TAMs. The color code indicates the correlation coefficient (Pearson r).

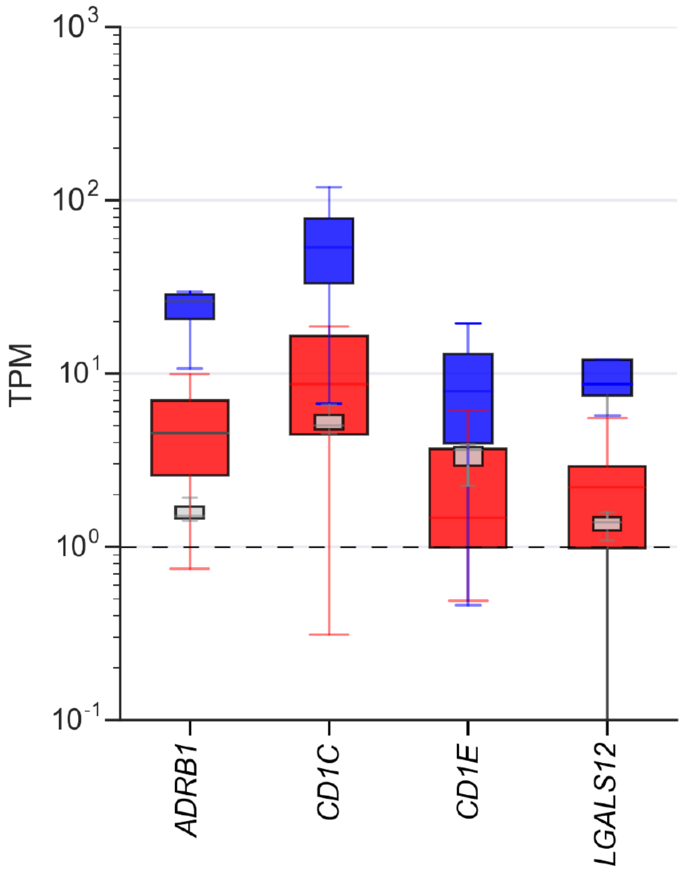

Supplementary Figure S3: Genes downregulated in TAMs (red) versus pMPHs (blue) and MDMs (grey). Boxes show the upper and lower quantiles and whiskers the 95% confidence intervals (RNA-Seq data).

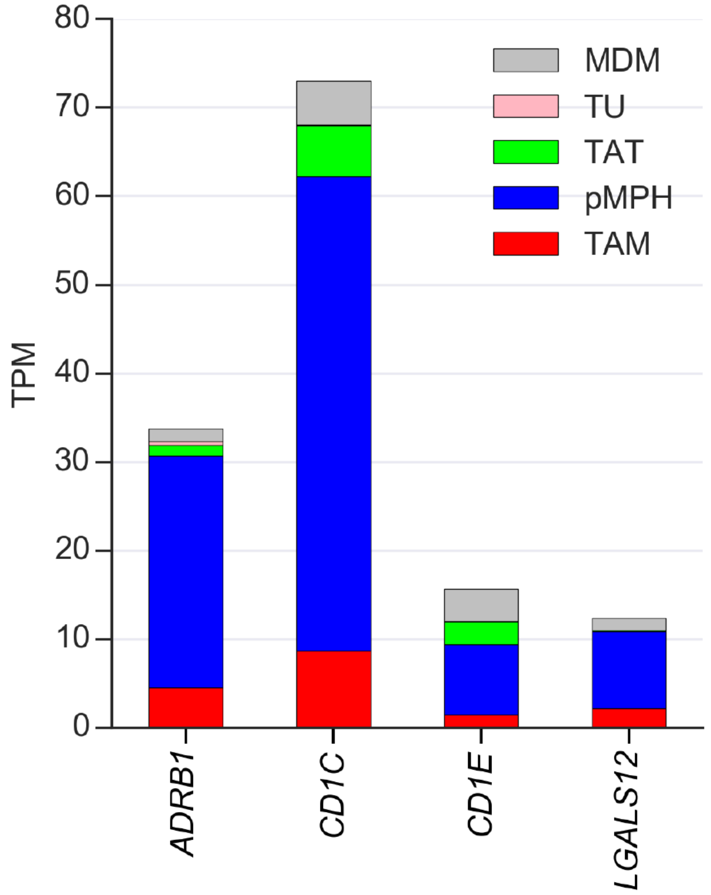

Supplementary Figure S4: Expression of genes downregulated in TAMs in different ovarian carcinoma-associated cell types, pMPHs and MDMs. The stacked boxes show the median expression values (TPM) of the genes in Supplementary Figure S3 for TAMs, pMPHs, MDMs, TATs and tumor cells.

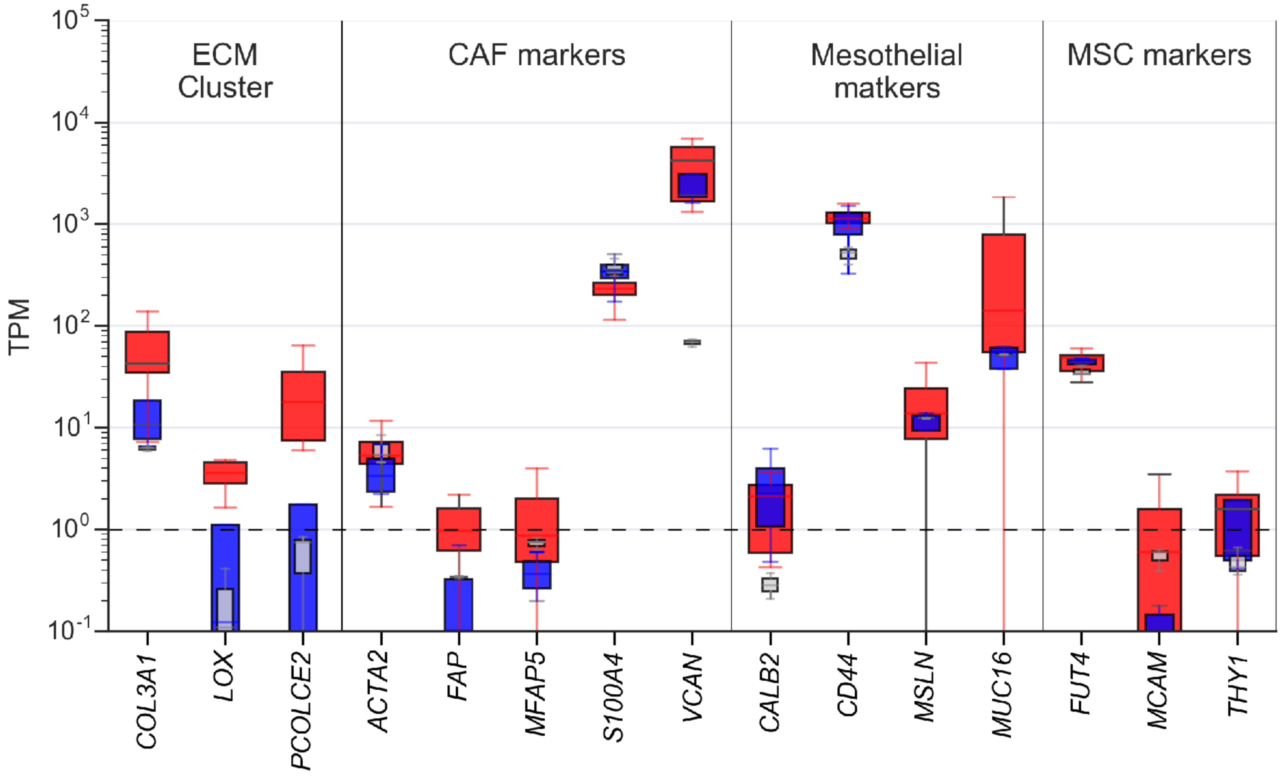

Supplementary Figure S5: Expression of CAF and mesothelial marker genes in TAM (red), pMPH (blue) and MDM (grey) samples. Boxes show the upper and lower quantiles and whiskers the 95% confidence intervals (RNA-Seq data).

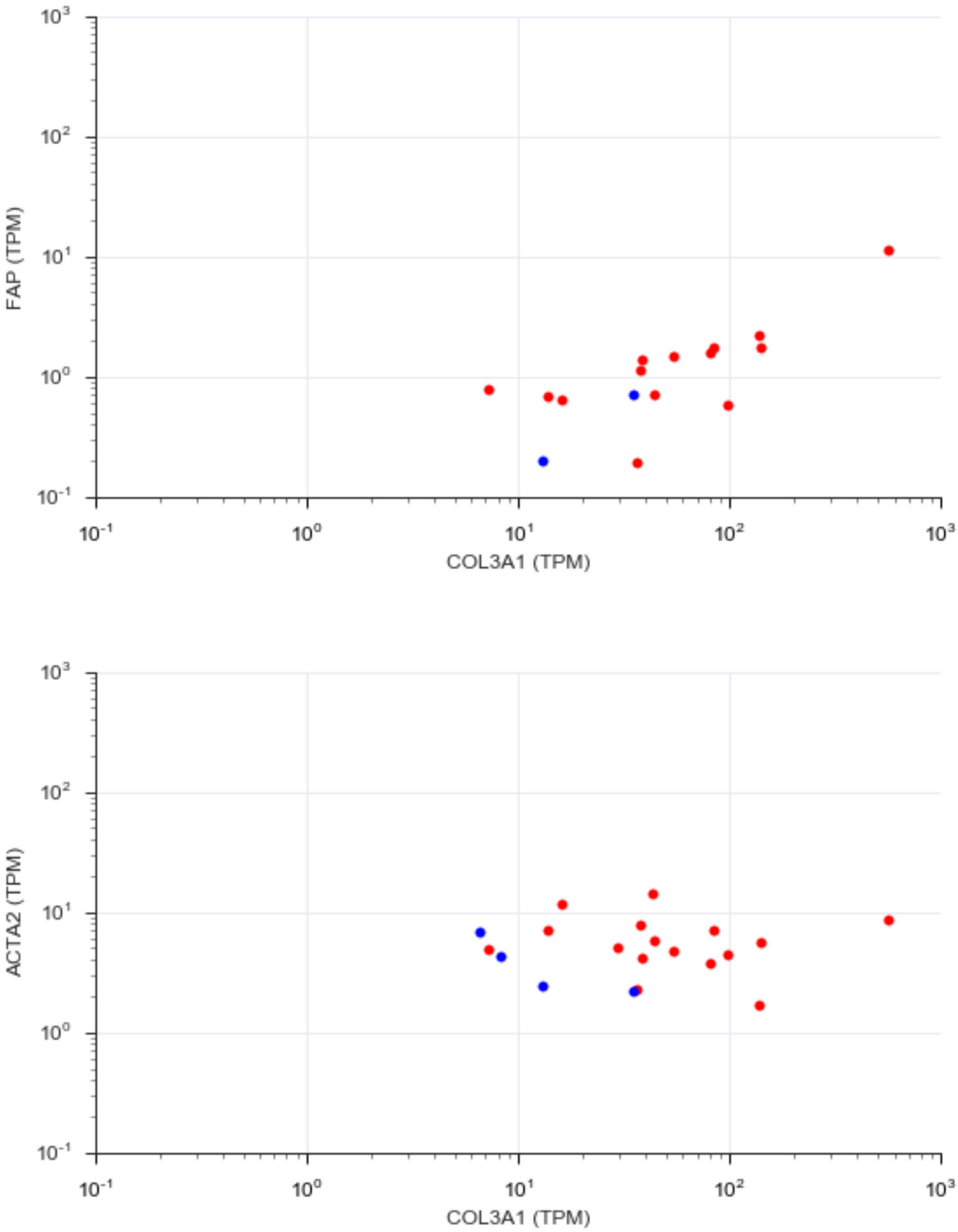

Supplementary Figure S6: CAF marker gene expression plotted versus the expression of the ECM signature gene *COL3A1*. Data represent TPM values. TAM: red; pMPH: blue. Two pMPH samples are missing from the upper plot due a lack of *FAP* expression (TPM = 0).

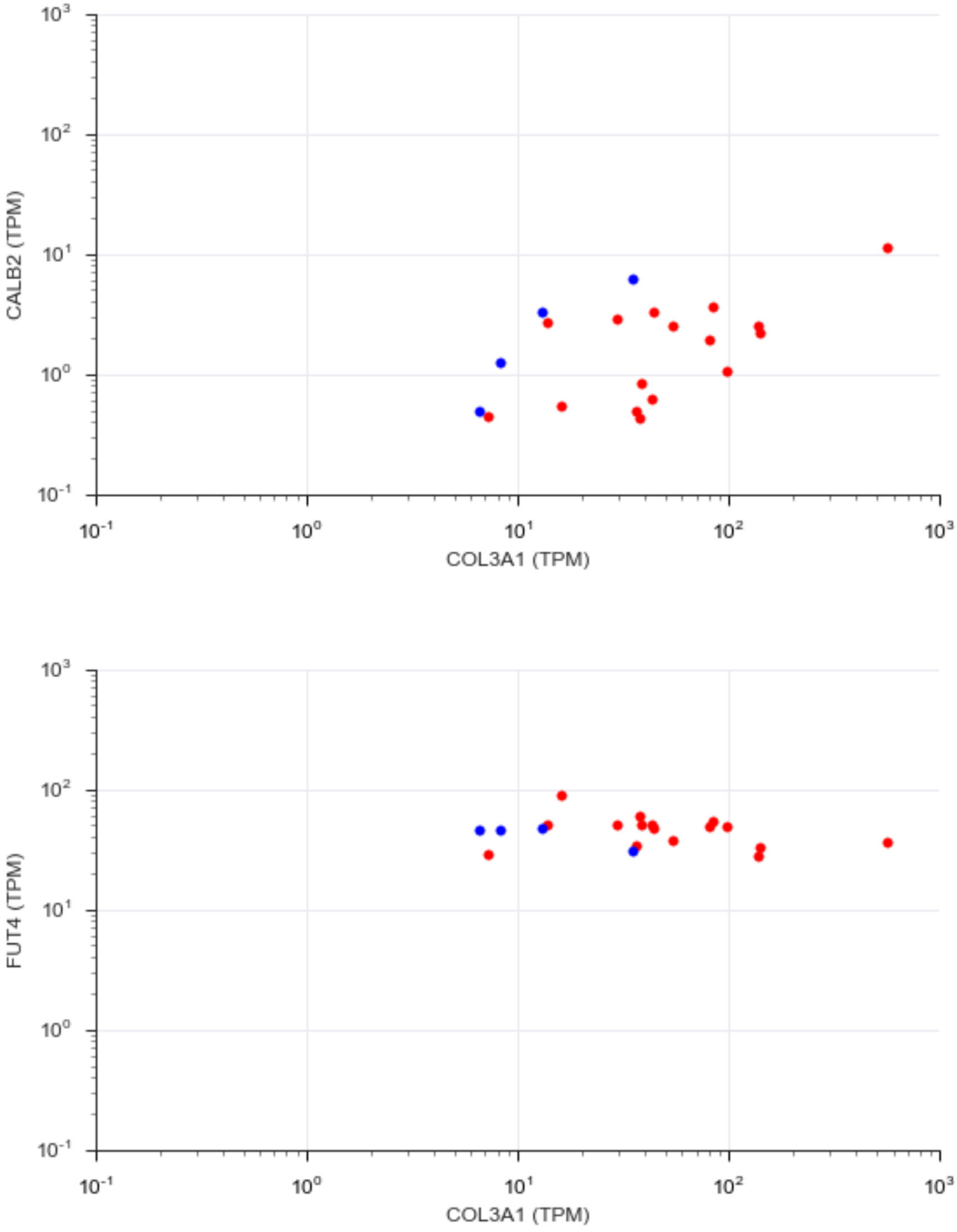

Supplementary Figure S7: MSC (top) and mesothelial (bottom) marker gene expression plotted versus expression of the ECM signature gene *COL3A1*. Data represent TPM values. TAM: red; pMPH: blue.

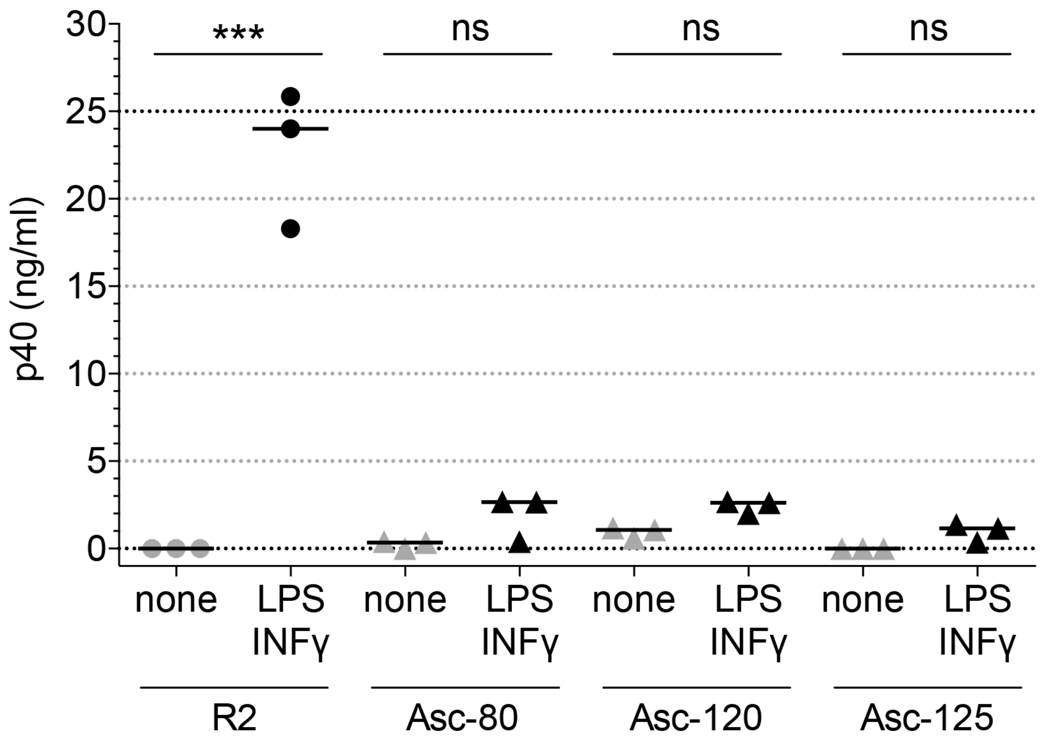

Supplementary Figure S8: Ascites-mediated inhibition of IL-12 (p40) secretion by MDMs. Data were obtained by ELISA as decribed in Figure 4. R2: RPMI medium with 2% human AB serum. Asc: MDMs in the presence of 3 different ascites samples during the differentiation period (6 days).

Supplementary Table S1: Genes regulated in TAMs versus pMPHs identified by edgeR

See Supplementary File 1

Supplementary Table S2: Genes regulated in TAMs versus MDMs identified by edgeR

See Supplementary File 1

Supplementary Table S3: GO term analysis of genes upregulated in TAMs versus pMPHs

See Supplementary File 1

Supplementary Table S4: Estimated tumor cell content of TAM samples*

See Supplementary File 1

Supplementary Table S5: Patient samples

See Supplementary File 1

Supplementary Dataset S1: RNA-Seq data for all genes (TPM)

See Supplementary Dataset 1

Supplementary Dataset S2: Genes upregulated in TAMs (TAM/pMPH, FC >3.0)

See Supplementary Dataset 1

Supplementary Dataset S3: Genes downregulated in TAMs (TAM/pMPH, FC <0.3)

See Supplementary Dataset 1
